# Microbiota composition and its impact on DNA methylation in colorectal cancer

**DOI:** 10.3389/fgene.2023.1037406

**Published:** 2023-08-08

**Authors:** Melva Gutierrez-Angulo, Maria de la Luz Ayala-Madrigal, Jose Miguel Moreno-Ortiz, Jorge Peregrina-Sandoval, Fernando Daniel Garcia-Ayala

**Affiliations:** ^1^ Departamento de Ciencias de la Salud, Centro Universitario de los Altos, Universidad de Guadalajara, Guadalajara, Jalisco, Mexico; ^2^ Doctorado en Genética Humana e Instituto de Genética Humana “Dr. Enrique Corona Rivera”, Departamento de Biología Molecular y Genómica, Centro Universitario de Ciencias de la Salud (CUCS), Universidad de Guadalajara, Guadalajara, Jalisco, Mexico; ^3^ Departamento de Biología Celular y Molecular, Centro Universitario de Ciencias Biológicas y Agropecuarias, Universidad de Guadalajara, Guadalajara, Jalisco, Mexico

**Keywords:** microbiota, DNA methylation, colorectal cancer, microbiome, DNA methyltransferase, tumor suppressor gene

## Abstract

Colorectal cancer is a complex disease resulting from the interaction of genetics, epigenetics, and environmental factors. DNA methylation is frequently found in tumor suppressor genes to promote cancer development. Several factors are associated with changes in the DNA methylation pattern, and recently, the gastrointestinal microbiota could be associated with this epigenetic change. The predominant phyla in gut microbiota are Firmicutes and Bacteroidetes; however, an enrichment of *Bacteroides fragilis*, *Fusobacterium nucleatum*, and *Streptococcus bovis*, among others, has been reported in colorectal cancer, although the composition could be influenced by several factors, including diet, age, sex, and cancer stage*. Fusobacterium nucleatum*, a gram-negative anaerobic bacillus, is mainly associated with colorectal cancer patients positive for the CpG island methylator phenotype, although hypermethylation in genes such as *MLH1*, *CDKN2A*, *MTSS1*, *RBM38*, *PKD1*, *PTPRT*, and *EYA4* has also been described. Moreover, *Hungatella hathewayi*, a gram-positive, rod-shaped bacterium, is related to hypermethylation in *SOX11*, *THBD*, *SFRP2*, *GATA5*, *ESR1*, *EYA4*, *CDX2*, and *APC* genes. The underlying epigenetic mechanism is unclear, although it could be implicated in the regulation of DNA methyltransferases, enzymes that catalyze the transfer of a methyl group on cytosine of CpG sites. Since DNA methylation is a reversible event, changes in gut microbiota could modulate the gene expression through DNA methylation and improve the colorectal cancer prognosis.

## 1 Introduction

Colorectal cancer (CRC) is a complex disease caused by interactions among genetic, epigenetic, and environmental factors. DNA methylation is frequently observed in tumor suppressor genes that promote cancer development. Several factors are associated with changes in DNA methylation patterns. Recently, the gastrointestinal microbiota have been associated with this epigenetic change. The predominant phyla in the gut microbiota are Firmicutes and Bacteroidetes. However, an enrichment of *Bacteroides fragilis*, *Fusobacterium nucleatum*, and *Streptococcus bovis*, among others, has been reported in CRC. Nevertheless, diet, age, sex, and cancer stage could influence the composition*. Fusobaterium nucleatum*, a gram-negative anaerobic bacillus, is mainly associated with CRC patients positive for the CpG island methylator phenotype. Notwithstanding, hypermethylation in genes such as *MLH1*, *CDKN2A*, *MTSS1*, *RBM38*, *PKD1*, *PTPRT*, and *EYA4* has also been described. Moreover, *Hungatella hathewayi*, a Gram-positive, rod-shaped bacterium, is related to hypermethylation in *SOX11*, *THBD*, *SFRP2*, *GATA5*, *ESR1*, *EYA4*, *CDX2*, and *APC* genes. The underlying epigenetic mechanism is unclear, although it could be implicated in regulation of DNA methyltransferases (DNMTs) , enzymes that catalyze the transfer of a methyl group on the cytosine of CpG sites. Since DNA methylation is a reversible event, changes in gut microbiota can modulate gene expression through DNA methylation and improve CRC prognosis.

CRC is the second leading cause of death and ranks fourth in incidence (Ferlay et al., 2020). CRC is a multi-step process characterized by the sequential accumulation of genetic and epigenetic changes that transform the normal epithelium into metastatic carcinoma (Li et al., 2021). Additionally, environmental factors have been found to be associated with this transformation, including consuming red and processed meat, alcohol, and tobacco; lack of physical activity; and microbiome composition (Katsaounou et al., 2022). Based on the morphological changes in colorectal tissue, CRC is divided into two categories: classical or adenoma–carcinoma sequences and alternative or serrated. In the adenoma–carcinoma sequence, the chromosomal (CIN) and microsatellite instability (MSI) has been associated with transforming normal tissue into adenocarcinoma tissue. The CIN pathway is found in 65%–70% of sporadic CRC cases and is characterized by mutations in oncogenes (*KRAS*) and tumor suppressor genes (*APC* and *TP53*), in addition to chromosomal aberrations (18q deletion). Furthermore, MSI is caused by defects in *MLH1*, *MSH2*, *MSH6*, and *PMS2*, which encode for mismatch DNA repair (MMR) proteins, and the MSI pathway is found in 15% of patients with sporadic CRC (Nguyen et al., 2020). In the alternative category, the serrated or sawtooth lesions include polyps, sessile, and traditional adenomas found in approximately 15% of CRC cases. The serrated pathway promotes these lesions, and the associated molecular changes include *BRAF* or KRAS mutations, MSI, microsatellite stability, and hypomethylation or hypermethylation in *MLH1* (De Palma et al., 2019; Nguyen et al., 2020). The CpG methylator island phenotype (CIMP) has been described in both categories (De Palma et al., 2019; Nguyen et al., 2020; Huang and Yang, 2022). This pathway is characterized by promoter hypermethylation of tumor suppressor genes. CIMP-positive tumors are identified by evaluating a panel comprising up to 16 genes (Jung et al., 2020). Frequently methylated markers used were *CACNA1G*, *IGF2*, *NEUROG1*, *RUNX3*, *SOCS1*, *CRABP1*, *MINT1*, *MINT2*, and *MINT31* (Jia et al., 2016). The CIMP pathway is associated with MSI through *MLH1* hypermethylation, and a high frequency is observed in serrated tumors and the proximal colon of older patients (Harada and Morlote, 2020).

## 2 Microbiota

The term “microbiota” refers to the composition and abundance of microorganisms. In contrast, the term “microbiome” is used to describe the whole habitat, including microorganisms, their genome, and environment, or is defined as a collection of genes and genomes of the microbiota (Marchesi and Ravel, 2015). Microbiota analysis has limitations owing to the difficulty in culturing microorganisms; however, the development of high-throughput sequencing of the 16S rRNA gene and shotgun metagenomics facilitates the identification of uncultured members of the gut microbiota (Milani et al., 2017; Kumar et al., 2023). The human gastrointestinal system contains the highest density of microbiota (10^11^–10^12^ per milliliter (Rinninella et al., 2019). The Unified Human Gastrointestinal Genome (UHGG) v2.0 estimates a total of 4,744 prokaryotic species (4,716 bacteria and 28 archaea) in the gut microbiome (Mitchell et al., 2020). Although everyone contains a unique gut microbiota, the predominant phyla are Firmicutes and Bacteroidetes, accounting for 90% of the total microbiota. *Clostridium* is the most frequent genus of Firmicutes, while *Bacteroides* and *Prevotella* are the most frequent genera of Bacteroidetes (Rinninella et al., 2019). Nevertheless, a large diversity of genera, such as *Pseudomonas*, *Streptococcus*, *Fusobacterium*, *Veillonella*, *Haemophilus*, *Neisseria*, *Porphyromonas*, *Collinsella*, *Faecalibacterium*, *Eubacterium*, *Ruminococcus*, *Peptococcus*, *Peptostreptococcus*, *Lactobacillus*, *Streptomyces*, and *Bifidobacterium*, has been reported in the gut microbiota (Sheng et al., 2019; Peery et al., 2021). Aruguman et al. (2011) analyzed 33 samples from European individuals using multidimensional cluster and principal component analyses. They identified different enterotypes based on the abundance of three principal genera: enterotype 1 (*Bacteroides*), enterotype 2 (*Prevotella*), and enterotype 3 (*Ruminococcus*). The function of *Bacteroides* and co-occurring *Parabacteroides* is protein or carbohydrate fermentation. In contrast, the function of *Prevotella* and co-occurring *Desulfovibrio* similar to that of *Ruminococcus* and co-occurring *Akkermansia* is mucin degradation. Almeida et al. (2021) compiled 204,938 genomes and 170,602,708 genes from the human gut microbiome to generate a UHGG catalog from prokaryote-isolated genomes and metagenome-assembled genomes (MAGs). The last method is used to infer a new genome from *de novo*-assembled contigs. They reported that the most representative bacterial species were *Agathobacter rectalis*, *Escherichia coli* D, *Bacteroides uniformis*, and the archaeal species, *Methanobrevibacter smithii*. Although a MAG was used, only two of the 25 most abundant bacteria were represented by the MAG. The microbiome is dynamic and influenced by age, anatomical region, diet, antibiotics, genetics, and others (Gomaa, 2020; Ruan et al., 2020). Different microbiome compositions through life stages have been reported and associated with diet diversity and inflammatory processes. Older adults (>65 years) have shown an abundance of Enterobacteriaceae compared to adults with mainly Bacteroidetes and Firmicutes*.* Moreover, a study of DNA of fecal microbiota of 69 samples (24 from individuals aged 105–109 years, 15 from individuals aged 99 to 104 years, 15 from individuals aged 65 to 75 years, and 15 from individuals aged 22 to 48 years) sequenced by Illumina revealed that the oldest individuals (105–109 years old) had enrichment of *Akkermansia*, *Bifidobacterium*, and Christensenellaceae bacteria (Biagi et al., 2016; Gomaa, 2020). Regarding the anatomical location, a transcriptome analysis of 20 samples collected from healthy people reported 20%–70% of similarity in samples from the upper gastrointestinal tract, including saliva, with predominant genera *Gemella*, *Veillonella*, *Neisseria*, *Fusobacterium*, *Streptococcus*, *Prevotella*, *Pseudomonas*, and *Actinomyces*, while 20%–90% of similarity was reported for the lower gastrointestinal tract with predominant genera *Faecalibacterium*, *Ruminococcus*, and *Bacteroides* (Vasapolli et al., 2019).

Pan et al. (2018) conducted a genome-wide analysis of intestinal epithelial cells in the small intestine of mice at different developmental stages. They found changes in microbiota-dependent methylation patterns early after birth, and the differentially methylated positions increased according to mouse development with 1,492 in 1-week-old mice, while in 4-week and 12/16-week were 132 and 217, respectively. Moreover, they found that the expression of Dnmt3a and Tet3, which are involved in DNA methylation and demethylation, respectively, was altered in 1- and 12/16-week-old mice. Analysis of differentially methylated genes revealed enrichment of genes associated with cellular proliferation, regeneration, and immune responses. The normal function of the gut microbiota is implicated in the metabolism of nutrients, xenobiotics, and drugs; protection against pathogens; structural support of the gastrointestinal tract; and immunomodulation (Jandhyala et al., 2015; Gomaa, 2020; Rebersek, 2021).

## 3 Microbiota and colorectal cancer

Controversial results regarding microbiota composition have been reported in patients with CRC. Higher abundances of *Butyrivibrio*, *Gemella*, *Akkermansia*, *Fusobacterium nucleatum*, *H. hathewayi*, *Parvimonas* spp., *Desulfovibrio*, *Streptococcus bovis*, *Bacteroides fragilis*, and *Bilophila wadsworthia* have been described in CRC (Dahmus et al., 2018; Sobhani et al., 2019; Wang, et al., 2020; Xia et al., 2020). Moreover, lower proportions of *Ruminococcus*, *Bifidobacterium*, *Eubacteria,* and *Lachnospira* have been reported (Sobhani et al., 2019). Different microbiota compositions have been described depending on the anatomical segment or cancer stage. The study conducted by Suga et al. (2022) found a significant reduction in *Clostridial* cluster XIVa and *Clostridial* cluster IX in sigmoid and right-sided colon cancers, respectively, as determined by the terminal restriction fragment length polymorphism analysis. Moreover, 16S rRNA gene sequencing revealed that Firmicutes were significantly dominant in right-sided colon cancer and *Verrucomicrobia* in sigmoid colon cancer. In proximal and distal segments, *Veillonella* and *Coprobacter* were more abundant in distal segments (Sheng et al., 2019). In CRC staging, *Alistipes* were abundant in patients with stage III compared with stage IV CRC (Sheng et al., 2019). Moreover, *Bacteroides fragilis* is associated with 3-year survival, whereas high levels of *F. nucleatum* are associated with poor survival in patients with metastatic CRC (Dahmus et al., 2018; Lee et al., 2018). An analysis of 118 patients with CRC, 140 with adenomas, and 128 healthy participants revealed enrichment of *Peptostreptococcus stomatis*, *Fusobacterium nucleatum*, *Parvimonas micra*, *Peptostreptococcus anaerobius*, and *Bacteroides fragilis* and depletion of *Coprobacter fastidiosus*, *Eubacterium ventriosum*, *Roseburia intestinalis*, and *Roseburia inulinivorans* in CRC compared with other groups. Moreover, the quantification of 97 metabolites showed an increase in 16 metabolites (L-alanine, glycine, L-proline, L-aspartic acid, L-valine, L-leucine, L-serine, myristic acid, phenyl lactic acid, oxoglutaric acid, L-phenylalanine, L-alpha-aminobutyric acid, phenylacetic acid, palmitoleic acid, 3-aminoisobutanoic acid, and norvaline), and one metabolite (butyric acid) was depleted in patients with CRC compared to healthy individuals (Coker et al., 2022).

Inflammation, production of mutagenic biomolecules, and, recently, a dysregulation of epigenetic mechanisms have been described as possible mechanisms of microbiota-induced colorectal carcinogenesis (Dahmus et al., 2018; Peery et al., 2021).

## 4 DNA methylation and microbiota

Epigenetics regulates gene expression without modifications in the nucleotide sequence composition, which can be inherited and reversible. Epigenetic mechanisms include DNA methylation, histone modification, and regulation by non-coding RNA (Portela and Esteller, 2010). DNA methylation is the most studied mechanism and is essential for tissue- or cell-specific gene expression regulation, silencing of retroviral elements, embryogenesis, genomic imprinting, and X chromosome inactivation (Portela and Esteller, 2010; Moore et al., 2013; Edwards et al., 2017). DNA methylation is a chemical modification in the fifth carbon of cytosine in CpG dinucleotides catalyzed by DNMTs and S-adenosylmethionine (SAM) as a cofactor. Three DNMTs are involved in DNA methylation: DNMT1 maintains the methylated state immediately after the DNA strand is replicated, and DNMT3A and DNMT3B promote *de novo* methylation, mainly during embryogenesis. Methylation is a reversible process controlled by DNMTs and demethylases. The demethylation process can be passive due to the lack of methyl addition during replication or active by TET proteins. In contrast, the last mechanism produces the intermediate metabolites 5-hydroxymethyl cytosine, 5-formylcytosine, and 5-carboxylcytosine, which are removed by base-excision repair (Dai et al., 2021). The methyl donor SAM, which is required for methylation reactions, is involved in folate metabolism. Folate metabolism is a multi-step process that produces 10-formyl tetrahydrofolate (THF), which is involved in purine synthesis, and 5–10 methylene THFs are required for thymidylate synthesis. Moreover, in one-carbon metabolism is generated 5-methylTHF, a metabolite that acts as a methyl donor for the conversion of homocysteine to methionine, which is subsequently metabolized to SAM, a cofactor of DNMT in the DNA methylation process (Liu and Ward, 2010).

The possible role of the microbiota in DNA methylation has been studied mainly in CIMP-positive tumors and *F. nucleatum*. CIMP is related to the hypermethylation of CpG islands found primarily in the promoters of suppressor genes, and a panel described previously was used for pathway identification. The CIMP status can be categorized into two groups: CIMP-positive or CIMP-negative, or into three groups: CIMP-high, CIMP-low, and CIMP-negative, depending on the number of methylated markers found in the colorectal tumor tissue (Jia et al., 2016). However, the microbiome and its association with CIMP have shown discordant results (Tahara et al., 2014; Ito et al., 2015; Mima et al., 2015; Park et al., 2017; Lee et al., 2018; Li J. et al., 2019). Tahara et al. (2014) identified high numbers of *F. nucleatum* and pan-*Fusobacterium* associated with CIMP positivity, and the phenotype was analyzed using seven markers (*ER*, *SFRP1*, *MYOD1*, *MGMT*, *SLC16A2*, *SPOCK2*, and *N33*) in CRC tissues. Moreover, concordant results have been reported by other authors who employed different markers for CIMP identification (Ito et al., 2015; Mima et al., 2015; Lee et al., 2018; Li J. et al., 2019; Ono et al., 2022), but no significant association was reported by Park et al., 2017. They analyzed eight markers for CIMP diagnosis (*MLH1*, *NEUROG1*, *CRABP1*, *CACNA1G*, *CDKN2A*, *IGF2*, *SOCS1*, and *RUNX3*), and only high *F. nucleatum* was significantly associated with *CDKN2A* methylation in CRC tissues with high MSI. Additionally, *MLH1* hypermethylation is associated with an abundance of *F. nucleatum* (Tahara et al., 2014; Ito et al., 2015; Mima et al., 2015). Mima et al. (2015) found no significance between LINE-1 (long interspersed nucleotide element-1) methylation and *F. nucleatum*. Moreover, a similar distribution of LINE-1 methylation levels and *F. nucleatum* was observed in negative and positive tumors for MSI-high (Hamada et al., 2018). Other species, such as *B. fragilis*, *Faecalibacterium prausnitzii*, and *E. coli*, have been analyzed, but no relationship with CIMP has been described (Li J. et al., 2019).

Few studies have explored the influence of the microbiota on gene-specific methylation. Sobhani et al. (2019) realized transferred human fecal samples from normal and CRC donors to germ-free mice, promoting aberrant crypt foci, microbiota dysbiosis, and DNA alterations in the murine colonic mucosa. In tissues, they reported hypermethylation in *SFRP1*,*2*,*3*, *PENK*, *NPY*, *ALX4*, *SEPT9*, and *WIF1* genes. However, only three genes (*WIF1*, *PENK*, and *NPY*) were selected for validation in the tissue, serum, and stool of patients with CRC, and hypermethylation in the blood was associated with microbiota. Moreover, hypermethylation in the *SFRP2* gene in tissues and blood has been associated with *Bilophila*. The protein encoded by *SFRP2* is a WNT pathway modulator that directly interacts with WNT ligands. Furthermore, the cumulative methyl index (CMI) was measured, and they found an abundance of *P. micra* in patients with a higher CMI in the blood (Sobhani et al., 2019). Xia et al. (2020) analyzed colonic mucosa, adenoma, and CRC tissues. They found promoter-wide methylation in *MTSS1*, *RBM38*, *PKD1*, and *PTPRT* related to *F. nucleatum* and *SOX11*, *THBD*, *SFRP2*, *GATA5*, and *ESR1* related to *H. hathewayi*, whereas *EYA4* was associated with both bacteria. Additionally, they analyzed *MLH1*, *APC*, *PTEN*, *P16* (*CDKN1A*), and *CDX1/2*, which are known driver genes of CRC, and found a correlation between CpG site methylation of *CDX2* and *MLH1* in *H. hathewayi* and *Streptococcus spp.*, respectively. Contrastingly, hypermethylation in *APC* was associated with both bacteria. Additionally, global DNA methylation (5-mC) was evaluated in the colonic cell lines NCM460, HCT116, and HT29 incubated with *F. nucleatum* and *H. hathewayi*, and a significant increase in global DNA methylation was reported in all of them (Xia et al., 2020).

Hypermethylated genes associated with *F. nucleatum* are *MLH1*, *CDKN2A*, *MTSS1*, *RBM38*, *PKD1*, *PTPRT*, and *EYA4*. *MLH1* is a tumor suppressor gene implicated in MMR and is frequently inactivated in CRC. The primary loss-of-function mechanism occurs through mutations, mainly in inherited CRC (Lynch syndrome); however, in sporadic CRC, approximately 19% of cases show hypermethylation of its promoter (Li et al., 2013). *CDKN2A* has shown a methylation frequency of 15% in CRC patients. The protein encoded by this gene is a cyclin-dependent kinase inhibitor; therefore, its hypermethylation increases cell proliferation (Bagci et al., 2016). *MTSS1* is a metastasis suppressor gene that is frequently hypermethylated in leukemia and prostate, gastric, and bladder cancers, excluding colorectal cancer, where there are no reports (Utikal et al., 2006; Yamashita et al., 2006; Xie et al., 2011; Chen et al., 2020; Grandits et al., 2021). Yu et al. (2010) analyzed 105 primary colon tumors and found hypermethylation in *TBX5* in 68% of cases. The transcription factor encoded by this gene upregulates to *MTSS1*; therefore, a *TBX5* hypermethylation also contributes to the inactivation of *MTSS1* (Yu et al., 2010). Hypermethylation induced by *F. nucleatum* could only be one of the mechanisms related to the inactivation of *MTSS1* since another process could be implicated. *RBM38* encodes for the mRNA 3′-untranslated region-binding protein to stabilize the transcripts, and hypermethylation has only been associated significantly in breast cancer with the *TP53* wild type (Léveillé et al., 2011). However, overexpression of RBM38 has been found in several cancers, including CRC (Tate et al., 2019). The protein encoded by *PKD1* belongs to the PKD family, which comprises PKD1, PKD2, and PKD3 members that regulate essential processes involved in the initiation and progression of cancer (Azoitei et al., 2018). Wei et al. (2014) analyzed expression in colon cancer tissues and found increased levels of PKD2 and a lower proportion of PKD3. However, PKD1 was not detected in either mRNA or protein but only in normal colon cells. They concluded that the loss of expression could result from epigenetic mechanisms, although they could not prove this. The *PTPRT* gene codes for phosphatase and is involved in cancer progression and hypermethylation in colorectal, lung, and head and neck cancers (Laczmanska et al., 2013; Peyser et al., 2016; Sen et al., 2020). Methylation analysis of The Cancer Genome Atlas data revealed that colon adenocarcinoma, head and neck squamous cell carcinoma, lung adenocarcinoma, and invasive breast carcinoma had *PTPRT* hypermethylation that was significantly correlated with the downregulation of mRNA expression, with CRC being the most frequently hypermethylated (Peyser et al., 2016). Finally, *EYA4* encodes for a phosphatase protein that functions in the DNA repair process, and the analysis has shown hypermethylation in CRC cell lines and approximately 90% of CRC patients (Kim et al., 2015; McInnes et al., 2017; Azuara et al., 2018). An analysis of epigenome-wide methylome of open chromatin in 12 CRC tissues revealed 2,187 differentially methylated regions, of which 66% located in 1,025 genes, with *EY4* being the most significant (Ishak et al., 2020).

The main hypermethylated genes associated with *H. hathewayi* were *SOX11*, *THBD*, *SFRP2*, *APC*, *GATA5*, *CDX2*, *ESR1* and *EYA4*. *SOX11* encodes a transcription factor, and its hypermethylation has been found in various malignancies (excluding CRC) and in the inflammatory rectal mucosa of ulcerative patients (Pugongchai et al., 2017; Tahara et al., 2017; Li X. et al., 2019; Shan et al., 2019). *THBD* encodes a receptor with an affinity for thrombin, and its hypermethylation is significant in gastric cancer-positive for *Helicobacter pylori* and CRC (Shin et al., 2010; Lange et al., 2012). Moreover, hypermethylation in *ESR1*, *CDX2*, *GATA5*, and *APC* has been reported in CRC patients (Dawson et al., 2014; Sahnane et al., 2015; Liang et al., 2017; Liu et al., 2017; Zhu et al., 2021). *ESR1* codes for estrogen receptor alpha, a transcription factor that regulates genes involved in differentiation and cell proliferation. However, the predominant form in colon tissue is estrogen receptor beta; therefore, its implication in CRC is unclear (Das et al., 2023). *CDX2* encodes a transcription factor that regulates genes in the intestinal epithelium (UniProt Consortium, 2021), and gene hypermethylation is associated with BRAF mutations and CIMP-high (Dawson et al., 2014). The protein produced by the *GATA5* gene is a transcription factor required for cardiovascular development. Nonetheless, in CRC, *GATA5* has been included in the CIMP panel because it is frequently methylated (Jia et al., 2016). Moreover, the protein encoded for the *APC* gene is involved in the WNT signaling pathway affected in 90% of sporadic CRC cases (Zhao et al., 2022).

## 5 Discussion

Recently, the role of epigenetic changes in tumor tissue induced by bacteria has been described, although there are few studies in this field. DNA methylation is associated with *F. nucleatum*, *Bilophila*, *P. micra*, *H. hathewayi*, and *Streptococcus* spp.

The most studied opportunistic pathogen is *F. nucleatum* which is found in several reservoirs and patients with CRC, and high levels have been associated with development, prognosis, and treatment response (Brennan and Garret, 2019). *Fusobacterium* is hypothesized to be associated with CRC through chronic infection and dysregulation of the expression of genes involved in the WNT pathway, immune system, and cell cycle (Gholizadeh et al., 2017; Brennan and Garret, 2019; Stokowa-Sołtys et al., 2021). *F. nucleatum* has been associated with CIMP+ and the promoter methylation of *MLH1*, *CDKN2A*, *MTSS1*, *RBM38*, *PKD1*, *PTPRT*, and *EYA4*, which are mutated and dysregulated in CRC according to the COSMIC database (Tate et al., 2019). CIMP+ tumors are characterized by hypermethylation in promoters of suppressor tumor genes, and sporadic CRC is positive for this phenotype up to 15%. Different molecular markers have been used for diagnosis, and *MLH1*, *GATA5*, and *CDKN2A* are occasionally included in the diagnosis panel (Jia et al., 2016; Park et al., 2017). The mechanisms of *F. nucleatum* that explain its role in DNA methylation are linked to inflammation and the generation of reactive oxygen species (ROS) (Koi et al., 2018). Although the mechanism of inflammation remains unclear, there is evidence of aberrant methylation associated with inflammatory cells in ulcerative colitis and gastric cancer, which are positive for *Fusobacterium* and *H. pylori*, respectively (Tahara et al., 2017; Koi et al., 2018). Regarding ROS, guanine is damaged to produce 7-8-dihydro-8-oxo-guanine, and CpG islands are highly dense in this nucleotide; therefore, the affected region recruits DNMTs, which silence the gene through hypermethylation (Koi et al., 2018). Moreover, *F. nucleatum* promotes the expression and activity of DNMT1 and DNMT3A in two CRC cell lines (HT29 and HCT116) and DNMT3B in the normal cell line, NCM460 (Xia et al., 2020). *Fusobacterium* and its relationship with DNA methylation could be due to butyrate fatty acids because high *Fusobacterium* abundance was significantly correlated with a decrease in 4-hydroxybutyric acid in CRC patients (Wang et al., 2020). 4-Hydroxybutyric acid is an intermediate in butyrate synthesis that regulates enzymes involved in DNA demethylation, methylation, histone acetylation, and methylation (Wang et al., 2022).

Only one study on *Bilophila* and SFRP2 methylation reported a methylation frequency of 66% in CRC patients associated with poorly differentiated tissues (Bagci et al., 2016; Sobhani et al., 2019; Yu et al., 2019). *Bilophila* and *Fusobacterium* are sulfate-reducing bacteria that produce hydrogen sulfide, a genotoxic compound that causes DNA damage (Dahmus et al., 2018). However, there is no evidence linking the alterations in DNA methylation to sulfide.

*Hungatella hathewayi*, a gram-positive, rod-shaped bacterium, is associated with hypermethylation in *SOX11*, *THBD*, *SFRP2*, *GATA5*, *ESR1*, *EYA4*, *CDX2*, and *APC* genes. *H. hathewayi* seems to increase the expression and nuclear activity of DNMT1 and DNMT3A in the CRC cell lines HT29 and HCT116 and the normal cell line NCM460 (Xia et al., 2020). Therefore, DNA methylation induced by this microorganism could occur through this pathway.

*Streptococcus spp*. are associated with *MLH1* and *APC* hypermethylation, genes frequently associated with CRC (Li et al., 2013; Liang et al., 2017; Zhu et al., 2021). *Streptococcus* species, such as *Streptococcus bovis* and *Streptococcus gallolyticus*, have been described in CRC, and their carcinogenic effect could be linked to increased production of inflammatory molecules; the subspecies *gallolyticus* upregulates β-catenin, a transcription factor involved in cell proliferation (Karpiński et al., 2022).

In addition, *WIF1*, *PENK*, and *NPY* hypermethylation has been associated with the microbiota; although no specific microorganism has been reported, a CMI was associated with *P. micra* (Sobhani et al., 2019). Hypermethylation in *WIF1*, *PENK*, and *NPY* is found in CRC. However, the roles of NPY and *PENK* in tumorigenesis are not clear, whereas that of WIF1 is related to the inhibition of the WNT pathway (Hu et al., 2018; Overs et al., 2021; Pulverer et al., 2021). *Parvimonas micra* is associated with high-grade tumors and the consensus molecular subtype 1 (defined by increased immune infiltration) in CRC patients (Löwenmark et al., 2022). Therefore, the possible mechanism of DNA methylation could be described for *Fusobacterium*.

Furthermore, the influence of the microbiota on the methylation of transcripts has been tested in animal models. Jabs et al. (2020) compared N^6^-methyladenosine mRNA in the cecal segment of conventional and germ-free mice and found 312 differentially methylated transcripts; moreover, when they transplanted the microbiota (derived from conventional mice) in germ-free mice, after 4 weeks of follow-up, they did not find differences in N^6^-methyladenosine compared to both groups of the study (Jabs et al., 2020).

**FIGURE 1:**
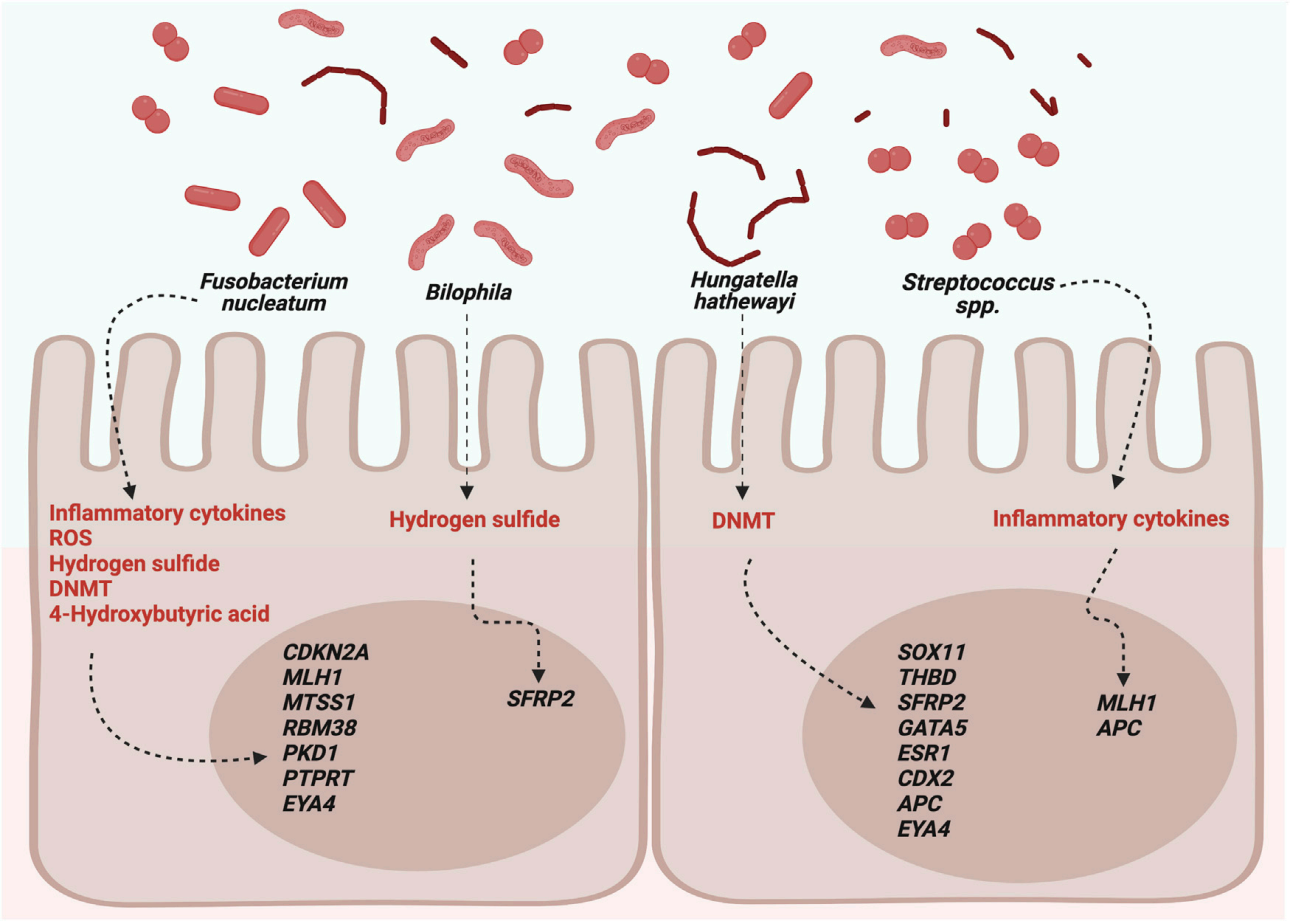
Possible roles of microbiota in DNA methylation. DNMTs, DNA methyltransferases; ROS, reactive oxygen species. Created with BioRender.com.

The main bacteria associated with DNA methylation and their possible mechanisms are shown in Figure 1.
